# MALDI-TOF MS for the Identification of Cultivable Organic-Degrading Bacteria in Contaminated Groundwater near Unconventional Natural Gas Extraction Sites

**DOI:** 10.3390/microorganisms5030047

**Published:** 2017-08-10

**Authors:** Inês C. Santos, Misty S. Martin, Doug D. Carlton, Catarina L. Amorim, Paula M. L. Castro, Zacariah L. Hildenbrand, Kevin A. Schug

**Affiliations:** 1 Department of Chemistry and Biochemistry, The University of Texas at Arlington, Arlington, TX 76019-0065, USA; ines.santos@uta.edu (I.C.S.); mistysmartin@mavs.uta.edu (M.S.M.); doug.carlton@uta.edu (D.D.C.J.); 2 Affiliate of the Collaborative Laboratories for Environmental Analysis and Remediation, The University of Texas at Arlington, Arlington, TX 76019-0065, USA; 3 CBQF—Centro de Biotecnologia e Química Fina—Laboratório Associado, Escola Superior de Biotecnologia, Universidade Católica Portuguesa/Porto, Rua Arquiteto Lobão Vital, Apartado 2511, 4202-401 Porto, Portugal; camorim@porto.ucp.pt (C.L.A.); plcastro@porto.ucp.pt (P.M.L.C.); 4 Biology Department and CESAM, University of Aveiro, Campus Universitário de Santiago, 3810-193 Aveiro, Portugal; 5 Inform Environmental, LLC, Dallas, TX 75206, USA

**Keywords:** mass spectrometry, hydraulic fracturing, groundwater, microorganism identification, bioremediation, toluene, chloroform

## Abstract

Groundwater quality and quantity is of extreme importance as it is a source of drinking water in the United States. One major concern has emerged due to the possible contamination of groundwater from unconventional oil and natural gas extraction activities. Recent studies have been performed to understand if these activities are causing groundwater contamination, particularly with respect to exogenous hydrocarbons and volatile organic compounds. The impact of contaminants on microbial ecology is an area to be explored as alternatives for water treatment are necessary. In this work, we identified cultivable organic-degrading bacteria in groundwater in close proximity to unconventional natural gas extraction. *Pseudomonas stutzeri* and *Acinetobacter haemolyticus* were identified using matrix-assisted laser desorption/ionization-time-of-flight-mass spectrometry (MALDI-TOF MS), which proved to be a simple, fast, and reliable method. Additionally, the potential use of the identified bacteria in water and/or wastewater bioremediation was studied by determining the ability of these microorganisms to degrade toluene and chloroform. In fact, these bacteria can be potentially applied for in situ bioremediation of contaminated water and wastewater treatment, as they were able to degrade both compounds.

## 1. Introduction

According to the United States Center for Disease Control [1], 15 million U.S. households rely on private household wells for drinking water. Therefore, groundwater, as a source of drinking water, should never be exposed to contamination of any kind. However, it can be subjected to contamination through different sources of human activity such as septic tanks, sanitary landfills, garbage dumps, concentrated animal feeding operations, mining and construction, fertilizers and pesticides, industrial products and wastes, household wastes and plumbing materials, and water treatment chemicals [2,3]. Contaminants such as heavy metals, pesticides, hazardous chemicals, and water-borne microorganisms may be released from these anthropogenic activities. They can reach the groundwater and cause great risks to humans who use this water as a source of drinking water.

Recently, increased concern about environmental safety and human health has called attention to the possible contamination of groundwater by unconventional oil and gas extraction. This includes hydraulic fracturing, which consists of the injection of fluids at high pressure (480–850 bars) into shale formations to fracture impermeable rock and release the trapped oil and gas [4]. Fluids, made of 98–99.5% water with the addition of sand and chemical additives [4,5,6], are injected under high pressure to fracture petroliferous strata and increase the mobility of sequestered hydrocarbons. These chemical additives include biocides to control microbial growth, friction reducers, oxygen scavengers to prevent corrosion, and acids [7]. After hydraulic stimulation, waste fluid returns to the surface due to the high pressure in the well. This fluid, referred to as flowback water, is wastewater that contains high levels of dissolved solids, salts, and fracturing chemicals and can be disposed of, treated, and/or reused. When the flowback finishes, fluid that is within the oil or gas-producing formation can be recovered and is called produced water; this has a high concentration of salt and contains harmful levels of metals and radioactivity [4,7]. All of the involved fluids, flowback and produced water, can be a source of contamination as they can enter the groundwater through spills on the surface while the well is stimulated, during transport and storage, or through faulty well casings [7].

The migration of natural gas constituents into the groundwater is another source of contamination that can occur from well stimulation activity, improper well casing, and/or poor on-site wastewater storage practices. Some studies have already been performed to understand the source of groundwater contamination near extraction sites [8,9,10,11,12]. In the Barnett shale of northern Texas, groundwater collected in close proximity to unconventional gas wells has been found to contain elevated levels of heavy metals, ethanol, and methanol [8], as well as organic solvents and chlorinated compounds [12]. In the study by Hildenbrand et al. [12], methanol and ethanol were detected in a great number of the water wells analyzed. No conclusions could be made about the source of these two alcohols as they can be produced in situ by bacteria but are also known to be used as anti-corrosive and gelling agents in unconventional oil and gas extraction.

Currently, little is known about the impact of hydraulic fracturing activities on the microbial ecology of groundwater. In fact, the presence of exogenous chemicals can alter microbial communities and these changes in population, whether increases or decreases, can be used as a tool to monitor the quality of groundwater and indicate possible contamination from anthropogenic activities, such as hydraulic fracturing. In spite of the presence of biocides in the fracturing fluids, studies [13,14,15,16] have shown that some bacteria are able to survive these conditions. During the drilling process, the chemicals used to open the soil can be released and contaminate the surroundings, including groundwater [17]. If the bacteria found in these waters can survive and proliferate in the presence of contaminants, their use for bioremediation can be evaluated. In this scenario, bacteria are not just an indicator of water quality but can also be used as a tool for water treatment. Some studies have been made in this field [18,19,20], to search for microorganisms that can degrade hazardous compounds. This alternative form of water treatment has tremendous value as pollutants can be degraded to less toxic compounds using natural biological activity. Also, bioremediation uses technologically simple techniques that can be carried out on site, so it is more economically advantageous when compared to traditional water treatment methods.

The identification of bacteria using matrix-assisted laser desorption/ionization (MALDI) time-of-flight mass spectrometry (TOF-MS) is an increasing area of interest. Several reviews have already been published that describe the use of this technique for microorganism identification (ID) along with other applications [21,22,23,24,25,26,27,28]. MALDI-MS allows the rapid, low-cost, and specific identification of microorganisms when compared to conventional microbial methods such as physiological, biochemical, and genomic methods. Microorganisms are placed in a target plate, where they are overlaid with a matrix solution that crystallizes with the sample and lyses the cells. The plate is placed in the instrument, where the proteins are ionized by laser pulses to form gas phase ions. The formed analytes are accelerated through the application of an electric field, to be subsequently separated by their differences in time-of-flight. This mass analysis provides a spectral fingerprint that is unique to the microorganism being analyzed. The organism is then identified by comparing its spectral profile to a reference database. Protein mass pattern spectra can be used to identify bacteria on the genus, species, and even the subspecies level. In spite of having clear advantages, this method still requires a cell culturing step to obtain pure cultures and enough bacterial cells for extraction and analysis. This might be considered a drawback, but it is also common to every other conventional identification method. In fact, it has been estimated that >99% of microorganisms observable in nature cannot be cultivated by standard techniques [29]. Thus, a large fraction of the diversity in an environment cannot be assessed using enrichment and isolation standard techniques. Nevertheless, the identification of cultivable bacteria using MALDI-TOF MS, in comparison with Sanger sequencing, is an easier and faster method, as it can be performed with individual colonies grown on plates, without the need for DNA extraction, amplification, and sequencing.

The novelty of this work lies in the identification of organic-degrading microorganisms present in groundwater potentially contaminated by unconventional oil and gas development. The prior studies performed in this field have only focused on the negative impacts of bacteria in unconventional oil and gas extraction activities, where bacteria were identified in hydraulic fluids [14,15,16] and produced water. This research is of extreme importance as it expands our understanding of the microbial ecology of sub-surface contamination events from anthropogenic processes. A secondary purpose of this research was to evaluate whether or not pertinent microorganisms detected in contaminated groundwater were able to degrade some possible organic contaminants, such as toluene and chloroform. As the bacteria can survive and grow in the presence of these chemicals, if they are also able to degrade them, they can be further utilized as an alternative for water bioremediation when, for example, a spill occurs.

## 2. Materials and Methods

### 2.1. Reagents

Tryptic soy agar (TSA), tryptic soy broth (TSB), and Mueller–Hinton broth were purchased from Difco (Detroit, MI, USA). Mass spectrometric grade water and acetonitrile were purchased from J.T. Baker (Phillipsburg, NJ, USA). Ethanol was purchased from Decon labs Inc. (Pure Ethanol 200 Proof, King of Prussia, PA, USA) and trifluoroacetic acid (TFA), chloroform, and toluene were purchased from Sigma-Aldrich (St. Louis, MO, USA). The MALDI matrix α-cyano-4-hydroxycinnamic acid (CHCA) was purchased from Sigma-Aldrich.

### 2.2. Groundwater Isolates

Water well samples were collected from private and public water supply wells as part of a reconnaissance analysis of groundwater quality throughout the Barnett shale region of northern Texas [12]. The presented samples were chosen due to their abnormal levels of organic pollutants, as shown in the Results section of this manuscript (Section 3.1). Five samples were collected from wells serving residential purposes, while two samples were collected from municipal or public water supply wells serving rural communities. In situ measurements were performed using a YSI Professional Plus multiparametric probe (Yellow Springs, OH, USA) and are presented in Table 1.

A volume of 100 µL of groundwater was plated in TSA and incubated at 37 °C for 24 h. The grown colonies were used for MALDI-TOF MS analysis and further confirmation tests were performed. Pure cultures were preserved at −80 °C in TSB supplemented with 15% (v/v) glycerol.

Bacteria quantification in water samples was performed according to the Standard Methods for Water and Wastewater Analysis [30]. Groundwater samples were diluted and inoculated on plate count agar followed by incubation at 37 °C. The grown bacteria were enumerated after 24 h.

### 2.3. MALDI-TOF MS

The sample preparation method used was the direct smear *plus* formic acid (on-target), according to the instrument manufacturer (Shimadzu Corporation, Kyoto, Japan). A colony was transferred with a sterile loop from the agar surface to the target plate and overlaid with 0.5 μL of 25% formic acid. After drying, 1.0 μL of CHCA matrix solution was deposited and allowed to dry.

The CHCA matrix solution (40 mg/mL) was prepared in 33% acetonitrile, 33% ethanol, 33% water, and contained a final concentration of 3% TFA.

Mass spectra were collected using the AXIMA-iD Plus MALDI-TOF Platform (Kratos Analytical, Manchester, UK). The mass spectrometer was operated in linear mode with a laser power of 57 and a detector voltage of 2600 V. Experiments were performed with 100 profiles per spectra and five shots per spot. The mass range was set at 2000–20,000 Da. Resolution of 500–600 was achieved for all the spectra at *m*/*z* around 7000. The equipment was calibrated with *Escherichia coli* DH5α, and the masses used were according to Freiwald and Sauer [31] and Ryzhov and Fenselau [32].

The identification of unknown bacteria in groundwater was performed by comparing protein mass spectral patterns with the Shimadzu Axima Confidence plus SARAMIS microorganism database.

### 2.4. Identification of the Isolated Strains

DNA from each isolate was extracted according to the heat-shock extraction method. Genomic DNA was further used for bacterial identification by 16S rRNA sequencing analysis using universal bacterial primers f27, f518, r800, and r1492, as previously described [33]. The 16S rRNA gene sequences were aligned with reference sequences available in the GenBank/EMBL/DDBJ database. The phylogenetic tree was constructed with the MEGA software (version 6.0, Tempe, AZ, USA) using the neighbor-joining method (Kimura two-parameter distance optimized criteria). The partial 16S rRNA gene sequences of the isolated strains were submitted to the GenBank database under accession numbers MF102101, *Acinetobacter* sp. strain A3, and MF102101, *Pseudomonas* sp. strain P3.

### 2.5. Biodegradation Studies of Toluene and Chloroform by the Isolated Bacteria

For the biodegradation studies, the bacteria were grown overnight in TSB. When at the log phase of growth, the bacteria were transferred to 50-mL conical flasks, each containing 50 mL of sterile mineral salts medium with toluene/chloroform (0.2% v/v). The experiment was carried out in duplicate and uninoculated flasks constituted the controls, accounting for abiotic losses. All flasks were incubated at 37 °C with 150 rpm and sampled over determined intervals of time (0, 1, 3, and 6 days). The mineral media was composed of (g/L): 0.2 MgSO_4_, 0.02 CaCl_2_, 1.0 KH_2_PO_4_, 1.0 K_2_HPO_4_, 1.0 NH_4_NO_3_, and 0.05 FeCl_3_. A headspace–gas chromatography (HS-GC) method with a flame ionization detector was used for the determination of toluene and chloroform [12]. Five milliliters were sampled and mixed with 1.00 mL of 0.25 M NaCl in a 20-mL screw-top headspace vial. Headspace operations were controlled with the AOC-5000 Plus headspace autosampler (Shimadzu Scientific Instruments, Inc., Columbia, MD, USA). The sample was incubated and agitated at 90 °C for 15 min. Seven hundred fifty microliters of vial headspace were sampled and injected. Separation and detection was achieved using a Shimadzu GC-2010 Plus (Shimadzu Scientific Instruments, Inc.) equipped with a Phenomenex ZB-BAC2 column (30 m × 0.32 mm × 1.2 μm) held at 40 °C for 4.5 min, then increased to 130 °C at 30 °C/min, and held for 2 min. A split ratio of 10:1 was used and the detector temperature was set at 200 °C.

The degradation rate constants (k, day^−1^) were calculated by assuming first-order kinetics, according to the following relationship: C = C_0_e^−kt^, where C_0_ and C are the concentrations at the start of the experiment and at time (t, day), respectively. The half-life of biodegradation (t_1/2_) was estimated from k using: t_1/2_ = ln2/k [34].

### 2.6. Antibiotic Susceptibility Tests

Pure streptomycin, piperacillin, oxacillin, vancomycin, ampicillin, and erythromycin were used. The antimicrobial agents were dissolved in water or methanol and diluted further with Mueller–Hinton broth media. The protocol to determine the antibiotic susceptibility was performed according to Andrews [35]. The following control strains were used for monitoring antimicrobial susceptibility testing: *Staphylococcus aureus Newman*, *Pseudomonas aeruginosa*, *Acinetobacter baumanii*, and *Escherichia coli K12*.

## 3. Results and Discussion

The goals of this work were: (1) to identify cultivable bacteria in contaminated groundwater near unconventional natural gas extraction sites; and (2) to study their application in water bioremediation. By doing so, novel information related to water quality could be discovered and an alternative to separation-based water treatment could be evaluated.

### 3.1. Water Quality

The first step was to assess the quality of the groundwater samples collected near gas extraction sites. Therefore, the determination of nutrients and different hazardous chemicals present in hydraulic fracturing fluids [36] was performed and is presented in Table 1 and Table 2.

TDS levels, shown in Table 1, averaged 675 mg/L, which exceeds the Environmental Protection Agency (EPA) TDS Drinking Water Maximum Contaminant Limit (MCL) of 500 mg/L. Six of the seven samples had pH measurements above 8.5, which is outside the pH range suggested by the EPA MCL (6.5–8.5).

Dichloromethane (DCM) was not quantifiable in three of the seven samples, while four samples (346, 369, 377, and 380) had an average concentration of 0.175 mg/L (Table 2), which exceeded the EPA MCL of 0.005 mg/L. Benzene was only detected in two of the seven samples and levels averaged 0.066 mg/L, which is also above its EPA MCL value of 0.005 mg/L. This is a concern as benzene is a known carcinogen [37]. According to the U.S. EPA, the recommended limit for chloroform in drinking water is 0.070 mg/L. Only one groundwater sample had a detectable value of chloroform at 2 mg/L, which exceeded that limit. This quantity is also of concern since chloroform is carcinogenic to animals [38]. For xylene, toluene, and ethylbenzene, all samples had concentrations below the EPA MCL of 10, 1, and 0.7 mg/L, respectively [39]. Acetonitrile, methanol, and propargyl alcohol were also found within the collected samples at a maximum concentration of 23, 45, and 22 mg/L, respectively. Drinking water standards do not currently exist for these three compounds.

The described values indicate that the collected groundwater samples were contaminated with different hazardous chemicals and should not be used as a drinking water source. Ingestion of these compounds can have negative effects on human health [37,38,39], including carcinogenesis.

### 3.2. Bacteria Identification by MALDI-MS

**Figure 1:**
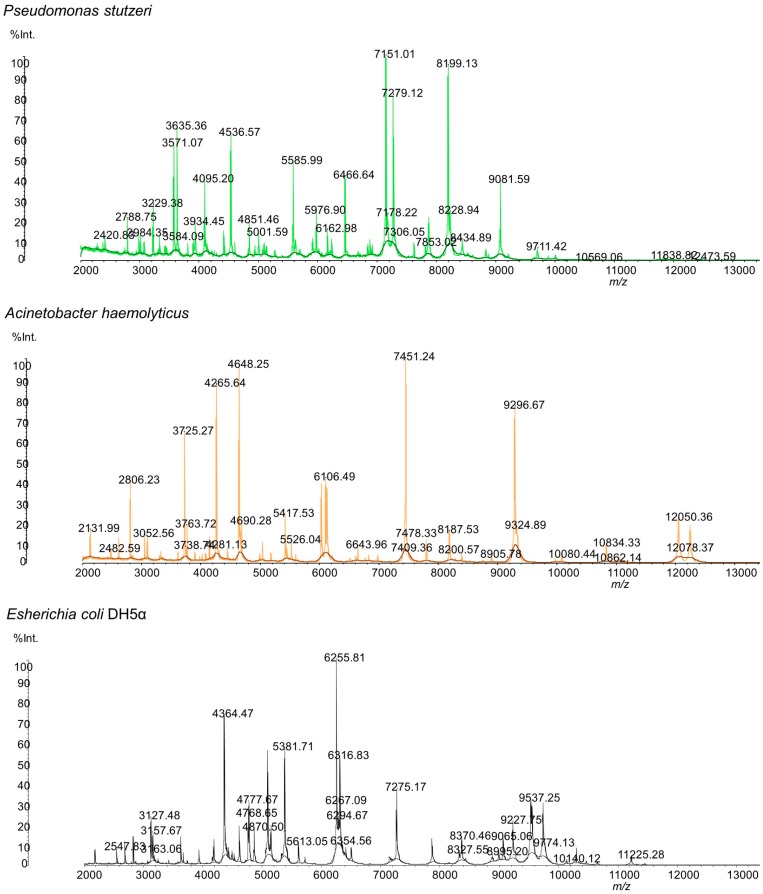
MALDI mass spectra of whole cells of the isolated bacteria and of the calibrant (*E. coli*) in CHCA matrix.

The water samples were plated in tryptic soy agar, as described in the Methods (Section 2.1). After plating the groundwater in the TSA plates and incubating the same at 37 °C for 24 h, two distinct colonies were observed. A distinct adherent colony with a wrinkled appearance was observed from all of the water samples, designated strain P3, and a smooth and pale colony was observed from two water samples (346 and 369) and designated strain A3. These bacteria colonies were transferred to a MALDI plate and analyzed. Results of the SARAMIS database search using the experimental masses were a 99.9% match for *Pseudomonas stutzeri* (strain P3) and *Acinetobacter haemolyticus* (strain A3). The MALDI mass spectrum of the whole cell of both microorganisms is shown in Figure 1.

The detected microorganisms exhibited very distinctive mass spectra, which enabled their differentiation and identification (Figure 1). Table 3 shows the *m*/*z* data and the respective peak intensities of each microorganism’s protein profile used for identification.

These findings were particularly interesting as, according to the literature, these two bacteria are usually found in the environment, specifically in soil and water [40,41]. In fact, *P. stutzeri* was previously identified in fluids used for hydraulic fracturing [14] and the authors suggested that this microorganism may be involved in the oxidation of alkane or aromatic compounds coupled to nitrate reduction. Its source may be the water used as a constituent of the hydraulic fluid, which can originate from different sources, such as surface water, groundwater, or recycled flowback [14]. The identified bacteria are not life-threatening to humans, although some cases have been reported where *P. stutzeri* caused infection [40,41,42,43] and *A. haemolyticus* caused bloody diarrhea [44,45].

According to the U.S. EPA [39], the heterotrophic plate count (HPC) number in non-filtered groundwater should be no more than 500 bacterial colonies per milliliter. Samples 314, 346, and 377 exceeded that value (Table 4). Therefore, it is not advisable for people to use this groundwater as a drinking water source without some form of pre-treatment, such as filtration or UV treatment. Interestingly, the water that presented lower bacteria counts for strain P3 is the one sample that had lower values of contaminants. It is plausible to hypothesize that the presence of these compounds enhances the growth of these microorganisms. Also, strain A3 was found in groundwater samples that presented lower concentrations of the detected contaminants. This may suggest that this microorganism is more susceptible to the presence of hazardous substances than strain P3. As such, strain A3 could potentially be used as a tool to monitor groundwater contamination if its presence is suppressed by the presence of contaminants.

### 3.3. Identification of the Strains by 16S rRNA Gene Sequencing

**Figure 2:**
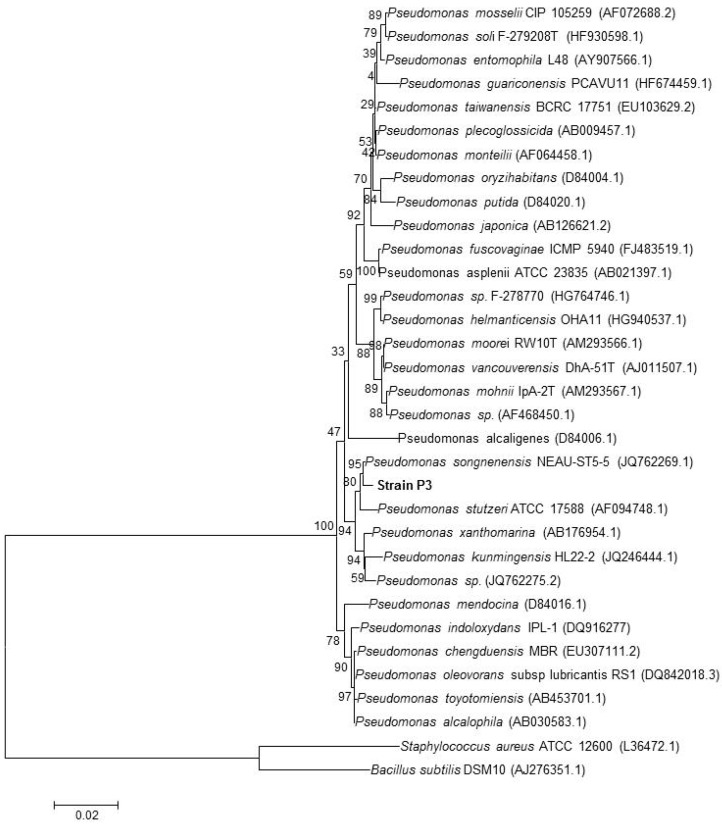
Neighbor-joining phylogenetic tree based on 16S rRNA gene sequences, showing the nearest neighbors of strain P3. *Bacillus subtilis* and *Staphylococcus aureus* type strains were used as an outgroup. Bootstrap values (expressed as percentages generated from 1000 replicates) greater than 50% are shown at branch points. GenBank accession numbers are given in parentheses. The bar represents 0.02 substitutions per site.

To validate the MALDI-TOF MS results, the microorganisms isolated from the groundwater samples were also identified by RNA sequencing. Phylogenetic analysis based on 16S rRNA gene sequences showed that strain P3 is a member of the genus *Pseudomonas* and formed a consistent cluster with *Pseudomonas songnenensis* (Acc. No. JQ762269.1; similarity = 99.6%), and *Pseudomonas stutzeri* (Acc. No. AF094748.1; similarity = 99.1) (Figure 2).

**Figure 3:**
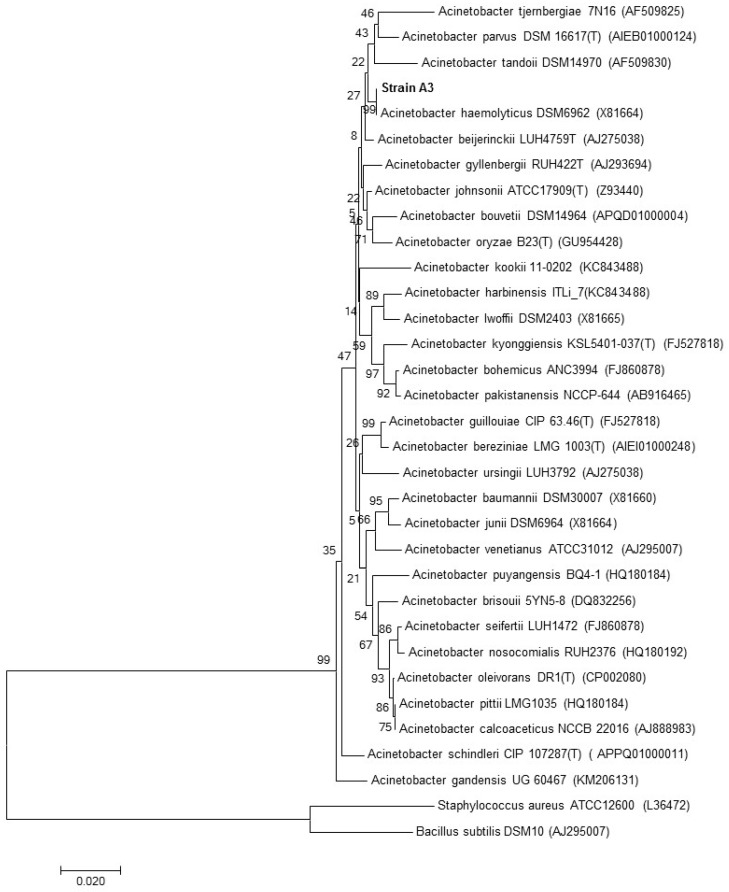
Neighbor-joining phylogenetic tree based on 16S rRNA gene sequences, showing the nearest neighbors of strain A3. *Bacillus subtilis* and *Staphylococcus aureus* type strains were used as an outgroup. Bootstrap values (expressed as percentages generated from 1000 replicates) greater than 50% are shown at branch points. GenBank accession numbers are given in parentheses. The bar represents 0.02 substitutions per site.

Phylogenetic analysis based on 16S rRNA gene sequences showed that strain A3 is a member of the genus *Acinetobacter* and formed a consistent cluster with *Acinetobacter haemolyticus* (Acc. No. JQ762269.1; similarity = 99.6%) (Figure 3).

### 3.4. Toluene and Chloroform Degradation Studies

Interestingly, homologous strains of the two detected microorganisms have been recognized as having the ability to degrade hydrocarbons [40]. Both *Pseudomonas* sp. P3 and *Acinetobacter* sp. A3 were present in the contaminated groundwater samples with higher concentrations of compounds, such as benzene, chloroform, and methanol. These data indicate that these bacteria are able to survive in water with high levels of contaminants. Therefore, a possible application in bioremediation was assessed.

**Figure 4:**
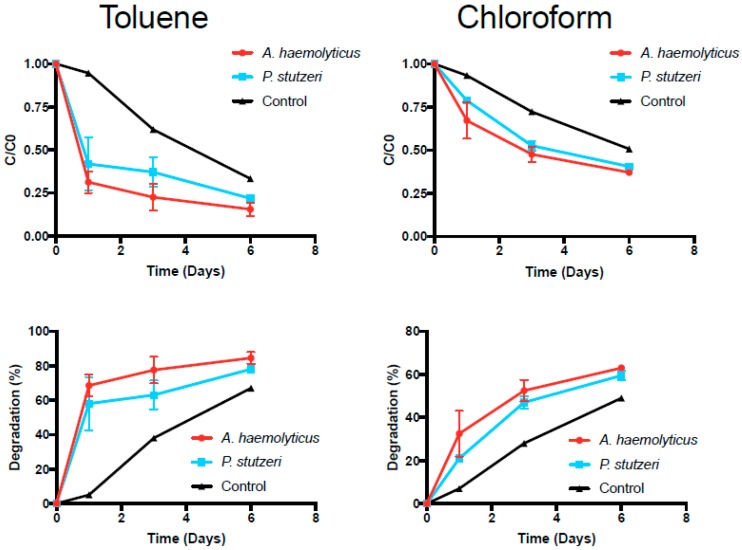
Degradation rates for toluene and chloroform. Data points represent the mean of two replicates, with the error bar representing the standard deviation.

It has been well established that certain forms of aerobic bacteria can use hydrocarbons as a source of carbon and energy [46]. Recent studies have been performed to determine the ability of *Pseudomonas* sp. and *Acinetobacter* sp. to degrade organic compounds such as aromatic hydrocarbons, alkanes, and halocarbons [47]. Our research was designed to assess whether or not the isolated bacteria are physiologically able to degrade some of the contaminants present in the groundwater samples. *Pseudomonas* sp. P3 and *Acinetobacter* sp. A3 were incubated in the presence of toluene and chloroform and the results obtained are presented in Figure 4.

Degradation rates of toluene and chloroform (Table 5) were notably different between the two experimental groups and the control group (the Kruskal–Wallis ANOVA yielded *p*-values of 0.050 and 0.025, respectively). The most notable differences between the degradation rates of the experimental groups and the controls were observed by day 1. Pairwise comparisons could not be performed to assess the statistical significance at each time point. Toluene degradation was faster than chloroform degradation. At the end of the six days, toluene concentrations were degraded to approximately 20% for both microorganisms, while chloroform concentrations were degraded to approximately 40%. The control also showed a decrease of toluene and chloroform concentrations over time, but not as significant as in the inoculated flasks, likely due to volatilization. In fact, it has been previously described that toluene-degrading microorganisms are also able to oxidize chloroform to carbon dioxide [48,49].

According to Cappelletti et al. [50], the toluene monooxygenase (ToMO) of *Pseudomonas stutzeri* OX1 can also oxidize chloroform. Several degradation pathways exist for toluene, as described by Jindrova et al. [51]. For example, this compound can be oxidized to benzyl alcohol and further to benzaldehyde and benzoate by the enzyme monoxygenase. The obtained results indicate that the isolated bacteria, *Pseudomonas* sp. P3 and *Acinetobacter* sp. A3, can potentially be used for bioremediation of contaminated groundwater or wastewater from unconventional oil and gas development. The studied biohazards can be degraded to less toxic compounds or harmless products such as carbon dioxide, water, and cell biomass.

### 3.5. Antibiotic Susceptibility

Isolated bacteria and those from an environmental source are not usually associated with an infectious process. However, the introduction of bacteria with wide antibiotic resistance in bioremediation is a very important subject. *Pseudomonas* spp. has the ability to acquire antibiotic resistance mechanisms [52] and *P. aeruginosa* is a well-described opportunistic pathogen that can cause severe infections in both healthy and immunocompromised individuals [53]. Of course, pathogenic bacteria should not be introduced into the environment; however, opportunistic pathogens such as *Pseudomonas* sp. can. These microorganisms, when introduced in the environment, can persist, proliferate, and spread, and can exchange their genetic material with other organisms. If these microorganisms are able to spread and proliferate, their presence may be innocuous or they can harm the ecology of the site [54]. Before using an opportunistic pathogen in bioremediation, it is necessary to determine the survival ability of the microorganism in different types of environments and the potential risks for the microbial community, environment, and human health.

As such, antibiotic susceptibility was evaluated for the isolated bacteria and antibiotics from different families were used. The obtained results are presented in Table 6. *Pseudomonas* sp. P3 and *Acinetobacter* sp. A3 were resistant to many of the tested antibiotics belonging to different families. This fact suggests that both microorganisms have a wide range of antibiotic resistance mechanisms. For *Pseudomonas* sp. P3, some mechanisms have already been described, such as alterations in outer membrane proteins and lipopolysaccharide profiles and the presence of β-lactamases [40], which can explain the resistance to streptomycin, vancomycin, and erythromycin. For *A. haemolyticus*, mechanisms such as the presence of β-lactamases and the production of aminoglycoside-modifying enzymes have also been described [55], which can explain its resistance to streptomycin, vancomycin, and ampicillin. As stated before, additional studies would be needed before using these microorganisms in bioremediation. It would be necessary to understand if, when applied to a certain site, these microorganisms will stay contained or will spread or exchange their genetic material. However, since these microorganisms are indigenous to the groundwater, it is unlikely that they will cause environmental damage. Nevertheless, their risks for human health should be thoroughly assessed.

## 4. Conclusions

The identification of cultivable bacteria in contaminated groundwater was performed using MALDI-TOF MS and the Shimadzu Axima Confidence plus the SARAMIS microorganism database. This methodology allowed for fast and easy identification of bacteria in water samples when compared to conventional ID methods. The identified bacteria, *Pseudomonas stutzeri* and *Acinetobacter haemolyticus*, are of environmental origin and are non-life-threatening to humans. These microorganisms have been reported as being able to degrade hydrocarbons such as chloroform, toluene, carbon tetrachloride, and 1,2-dichloroethane. Therefore, degradation studies were performed to determine if the isolated bacteria were able to degrade toluene and chloroform. In fact, both *Pseudomonas stutzeri* and *Acinetobacter haemolyticus* were able to degrade toluene and chloroform, and higher degradation rates were observed for toluene. These bacteria can potentially be used for bioremediation of contaminated water and wastewater obtained from unconventional oil and gas development. Subsequent studies can also be performed to apply the isolated bacteria for the degradation of other contaminants such as benzene and xylene. Furthermore, ex situ bioremediation studies could be performed to understand if the isolated microorganisms would still be able to degrade the compounds when placed in the actual contaminated groundwater and by varying physical conditions such as temperature.

Additionally, it will be necessary to understand if, when used for in situ bioremediation, these microorganisms pose a risk to human health and the indigenous microbial communities. Both microorganisms were resistant to many of the tested antibiotics, suggesting that they already have a wide range of antibiotic resistance mechanisms. Furthermore, it will be interesting to explore if exposure to pollutants may alter the bacterial responses and their virulence. 

## Figures and Tables

**Table 1:** Basic water quality analysis result and target use of water well investigated in this study.

Sample ID	Depth (ft)	Temp (°C)	DO (mg/L)	Specific Conductance (mS/cm)	TDS (mg/L)	Salinity (mg/L)	pH	Water Use
314	500	21.1	29.2	1.01	657	0.50	9.04	Residential
316	350–400	17.4	3.66	0.77	501	0.38	8.78	Residential
346	-	19.8	1.23	1.01	657	0.50	8.94	Residential
369	300	22.3	1.70	0.97	631	0.48	8.98	Residential
377	1580	30.1	1.56	1.11	722	0.55	8.58	Municipal
380	3370	31	1.57	1.52	988	0.74	8.08	Municipal
526	735	25.8	1.26	0.88	572	0.43	9.42	Residential

DO: dissolved oxygen; TDS: total dissolved solids.

**Table 2:** Concentrations (mg/L) of constituents in groundwater samples [12].

(mg/L)																	
Sample ID	TOC	IC	TN	MeOH	EtOH	DCM	Propargyl Alcohol	Acetone	ACN	Ethyl Acetate	Chloroform	Cyclo-hexane	Benzene	Toluene	Ethyl Benzene	*m,p-*Xylene	*o-*Xylene
314	0.1	91.2	0.5	44.6	0.874	0.000	0.000	0.740	23.0	1.14	0.000	0.017	0.000	0.000	0.007	0.009	0.017
316	0.2	94.6	0.5	2.17	7.03	0.000	22.1	0.331	2.57	1.19	0.000	0.014	0.083	0.000	0.002	0.000	0.023
346	0.1	0.1	0.5	0.00	0.000	0.216	0.000	0.000	0.037	0.009	2.14	0.000	0.000	0.000	0.000	0.000	0.009
369	0.1	0.1	0.5	0.00	0.000	0.136	0.000	0.000	0.028	0.000	0.000	0.014	0.000	0.000	0.000	0.003	0.010
377	0.4	85.8	0.5	0.00	3.71	0.203	0.000	0.178	1.90	6.41	0.000	0.000	0.000	0.000	0.012	0.005	0.006
380	2.2	119	0.7	0.00	0.413	0.144	0.000	0.026	0.019	0.000	0.000	0.013	0.048	0.051	0.007	0.000	0.010
526	0.0	89.4	0.6	0.00	0.000	0.000	0.000	0.000	0.031	0.039	0.000	0.000	0.000	0.001	0.000	0.000	0.005

TOC, total organic carbon; IC, inorganic carbon; TN, total nitrogen; MeOH, methanol; EtOH, ethanol; DCM, dichloromethane; ACN, acetonitrile.

**Table 3:** Masses derived from *A. haeomlyticus*, *P. stutzeri*, and *E. coli* DH5α preparations and their respective intensities.

*A. haemolyticus*		*P. stutzeri*		*E. coli*	
*m*/*z*	Intensity	*m*/*z*	Intensity	*m*/*z*	Intensity
2131.99	4,269,529	2420.83	2,147,092	2547.83	2,131,223
2482.59	1,506,536	2788.75	4,242,038	3127.48	5,397,132
2806.23	10,347,161	2984.35	3,109,296	3157.67	2,467,099
3052.56	3,607,775	3229.38	6,287,977	3163.06	1,425,940
3725.27	15,720,611	3571.07	12,904,611	**4364.47**	26,901,980
3738.74	1,824,080	3584.09	3,649,719	4768.65	5,157,224
3763.72	4,437,623	3635.36	11,766,971	4777.67	4,935,549
4265.64	30,881,306	3934.45	3,198,564	4870.50	3,468,297
4281.13	3,399,151	4095.20	6,913,595	**5381.71**	14,711,287
4648.25	27,630,984	4536.57	12,977,799	5613.05	2,307,677
4690.28	5,806,014	4851.46	6,236,720	**6255.81**	24,465,422
5417.54	6,592,444	5001.59	4,327,399	6267.09	5,618,275
5526.04	3,239,353	5585.99	9,931,623	6294.67	6,707,362
6106.49	14,694,100	5976.90	6,644,226	**6316.83**	13,191,460
6643.96	2,894,466	6162.98	3,457,942	6354.56	1,996,031
7409.36	2,194,172	6466.64	10,736,716	**7275.17**	11,564,301
7451.24	42,138,924	7151.01	30,722,710	7721.46	89,658.7891
7478.33	6,692,804	7178.22	11,632,331	8327.55	2,868,725
8187.53	5,622,694	7279.12	23,694,890	**8370.46**	7,628,921
8200.57	2,720,580	7306.05	3,114,205	9065.06	5,520,206
8905.78	1,443,282	7853.02	3,044,757	9227.75	9,474,368
9296.67	45,184,156	8199.13	28,981,870	9537.25	14,085,582
9324.89	10,356,339	8228.94	12,505,299	9555.28	15,322,127
10,080.44	1,686,673	8434.89	5,943,598	9741.23	15,633,452
10,834.33	5,093,954	9081.59	14,799,761	**10,140.12**	1,406,545
10,862.14	769,789	9711.42	3,061,340	10,653.57	863,826
12,050.36	14,516,812	10,569.06	527,712	11,225.28	2,137,546
12,078.37	3,561,288	11,838.82	1,172,665	11,452.91	484,301
		12,473.59	939,518		

Bold values represent the masses used for mass signal calibration.

**Table 4:** Bacteria quantification in groundwater samples. SD, standard deviation (*n* = 2).

Sample ID	Strain P3 cfu/mL ± SD	Strain A3 cfu/mL ± SD
314	9.2 (± 0.6) × 10^3^	-
316	2.0 (± 0.2) × 10^2^	-
346	1.68 (± 0.009) × 10^3^	21 ± 5
369	20 ± 3	3 ± 1
377	2.1 (± 0.1) × 10^3^	-
380	55 ± 9	-
526	110 ± 1	-

**Table 5:** Degradation rate constant (*k*) and half-life (t_1/2_) for degradation of toluene and chloroform by *Acinetobacter* sp. A3 and *Pseudomonas* sp. P3.

Bacteria	Toluene		Chloroform	
	*k* (day^−1^)	t_1/2_ (day)	*k* (day^−1^)	t_1/2_ (day)
*Acinetobacter* sp. A3	0.67 ± 0.12	1.41 ± 0.24	0.27 ± 0.04	2.96 ± 0.12
*Pseudomonas* sp. P3	0.50 ± 0.15	1.90 ± 0.31	0.20 ± 0.00	3.66 ± 0.03

**Table 6:** Antibiotic sensitivities of the isolated bacteria.

Family	Antibiotic	*Pseudomonas* sp. P3	*Acinetobacter* sp. A3
Aminoglycoside	Streptomycin	R	R
β-lactam, extended spectrum penicillin	Piperacillin	S	S
β-lactam, semisynthetic penicillin	Oxacillin	S	S
Glycopeptide	Vancomycin	R	R
β-lactam, extended spectrum penicillin	Ampicillin	S	R
Macrolide	Erythromycin	R	R

R, resistant; S, sensitive.
